# Inhibition of Tunneling Nanotube (TNT) Formation and Human T-cell Leukemia Virus Type 1 (HTLV-1) Transmission by Cytarabine

**DOI:** 10.1038/s41598-018-29391-w

**Published:** 2018-07-24

**Authors:** Maria Omsland, Cynthia Pise-Masison, Dai Fujikawa, Veronica Galli, Claudio Fenizia, Robyn Washington Parks, Bjørn Tore Gjertsen, Genoveffa Franchini, Vibeke Andresen

**Affiliations:** 1 Centre for Cancer Biomarkers (CCBIO), Precision Oncology Research Group, Department of Clinical Science, University of Bergen, Bergen, Norway; 2 Animal Models and Retroviral Vaccines Section, Vaccine Branch, National Cancer Institute, National Institutes of Health, Bethesda, Maryland USA; 3 Department of Pathophysiology and Transplantation, University of Milan, Milan, Italy; 4 Department of Internal Medicine, Haukeland University Hospital, Bergen, Norway

## Abstract

The human T-cell leukemia virus type 1 (HTLV-1) is highly dependent on cell-to-cell interaction for transmission and productive infection. Cell-to-cell interactions through the virological synapse, biofilm-like structures and cellular conduits have been reported, but the relative contribution of each mechanism on HTLV-1 transmission still remains vastly unknown. The HTLV-1 protein p8 has been found to increase viral transmission and cellular conduits. Here we show that HTLV-1 expressing cells are interconnected by tunneling nanotubes (TNTs) defined as thin structures containing F-actin and lack of tubulin connecting two cells. TNTs connected HTLV-1 expressing cells and uninfected T-cells and monocytes and the viral proteins Tax and Gag localized to these TNTs. The HTLV-1 expressing protein p8 was found to induce TNT formation. Treatment of MT-2 cells with the nucleoside analog cytarabine (cytosine arabinoside, AraC) reduced number of TNTs and furthermore reduced TNT formation induced by the p8 protein. Intercellular transmission of HTLV-1 through TNTs provides a means of escape from recognition by the immune system. Cytarabine could represent a novel anti-HTLV-1 drug interfering with viral transmission.

## Introduction

Worldwide, it is estimated that at least 5–10 million people are infected with human T-cell leukemia virus type-1 (HTLV-1), the first human oncogenic retrovirus discovered^1–4^. While most infected individuals remain life-long asymptomatic carriers, after a latency of several decades, approximately 3–5% develops an aggressive adult T-cell leukemia/lymphoma (ATL) and 1–4% a neurodegenerative condition HTLV-1 associated myelopathy (HAM)/tropical spastic paraparesis (TSP)^1–6^. HTLV-1 infections have additionally been associated with inflammatory conditions such as uveitis, bronchoalveolitis and arthritis, Sjögrens syndrome and polymyostis^7–10^.

*In vivo*, HTLV-1 has been found in different cell types such as CD4^+^ T-cells, CD8^+^ T-cells, dendritic cells, macrophages, B-cells, endothelial cells and monocytes^11–21^. However, only CD4^+^ memory T-cells undergo HTLV-1-dependent cellular transformation and is therefore considered the main cellular target of this virus^22,23^. For viral infection, Glucose Transporter 1 (GLUT1), Heparan Sulfate Proteoglycans (HSPG) and Neuropilin 1 (NRP-1) have been identified as the HTLV-1 receptor complex important for viral binding and cellular entry^24,25^. However, in contrast to HIV-1, cell-free HTLV-1 particles in the plasma of HTLV-1 infected individuals are almost undetectable and, except in dendritic cells, cell-free infections are very inefficient^26^. Therefore cell-to-cell contacts are considered the main and most efficient method of HTLV-1 transmission and spread^26–29^. Thus, mechanisms to reduce cell-to-cell transmission have the potential to significantly reduce viral burden. Two different cell-to-cell contacts have been described in HTLV-1 transmission. The virological synapse (VS) is defined as a virus induced tight cell-to-cell interaction creating a synaptic intercellular cleft allowing viral budding and transmission. This causes a viral induced polarization of the microtubule-organizing center (MTOC) in the donor cell towards the virological synapse^30,31^. Viral biofilms, consisting of extracellular matrix and lectins, have been discovered on the surface of HTLV-1 infected cell lines^32^. The HTLV-1 particles are concentrated and protected by the biofilms and transmitted to the target cell^32^. For long distance cell-to-cell interactions we previously reported viral transmission in T-cells by cellular conduits, a process enhanced by the HTLV-1 encoded protein p8^33,34^. The p8 protein is generated by a two-step proteolytic cleavage of the p12 protein^33^, encoded by open reading frame 1 (*orf-I*) in the HTLV-1 genome, previously demonstrated to be involved in viral persistence^35–37^. First, p12 is cleaved between amino acids in position 9 and 10 and then between amino acids 29 and 30, where the nature of the amino acids surrounding these cleavage sites determines the efficiency of cleavage and polymorphism has been found in HTLV-1 infected individuals^33,36^. Interestingly, also the p8 protein itself is transferred through cellular conduits to neighboring cells^34,38^. Transmission of HTLV-1 through these types of cell-to-cell contacts could provide protection from recognition by the immune system^34^.

In the present study, we further examined long-distance cell-to-cell interactions utilized by HTLV-1 expressing T-cells and found that these infected cells form tunneling nanotubes (TNTs), often containing the viral proteins Tax and Gag, between T-cells as well as monocytes. TNTs are thin (50–200 nm in diameter) and long (5–100 µm) cell-to-cell interconnecting structures lacking contact with the substratum, embedded by plasma membrane containing filamentous actin (F-actin)^39^. A variety of immune cells interact through TNTs including natural killer (NK) cells, macrophages, T-cells and B-cells^40–42^. TNTs have also been reported in HIV-1 infected T-cells and macrophages, in influenza A infected cells and cells infected by bovine herpesvirus 1^43–48^. In a recent study we demonstrated that the pyrimidine nucleoside analog cytarabine reduced NF-κB activation and TNT formation in acute myeloid leukemia (AML) cells^49^. Here we find that cytarabine treatment also resulted in reduced number of TNTs in HTLV-1 cells. In MT-2 cells, cytarabine reduced expression of Tax, Gag and viral production and caused a decrease in viral transmission from MT-2 cells as well as primary HTLV-1 infected CD4^+^ cells to uninfected cells. By sequencing of *orf-I* in the HTLV-1 infected cell lines included in this study we found that the MT-2 cells, demonstrating the highest amount of TNTs, contained the *orf-I* isoform N26, earlier demonstrated to express mostly the p8 protein^36^. Expression of orf-I-N26 (p8) in Jurkat cells enhanced TNT formation whereas cytarabine treatment reduced TNT numbers, however, not influencing p8 expression. Thus, our work shows that cytarabine could have possible therapeutic effects to reduce HTLV-1 transmission by reduced communication of HTLV-1 infected cells with uninfected cells.

## Results

### HTLV-1 expressing cells form tunneling nanotubes (TNTs)

**Figure 1:**
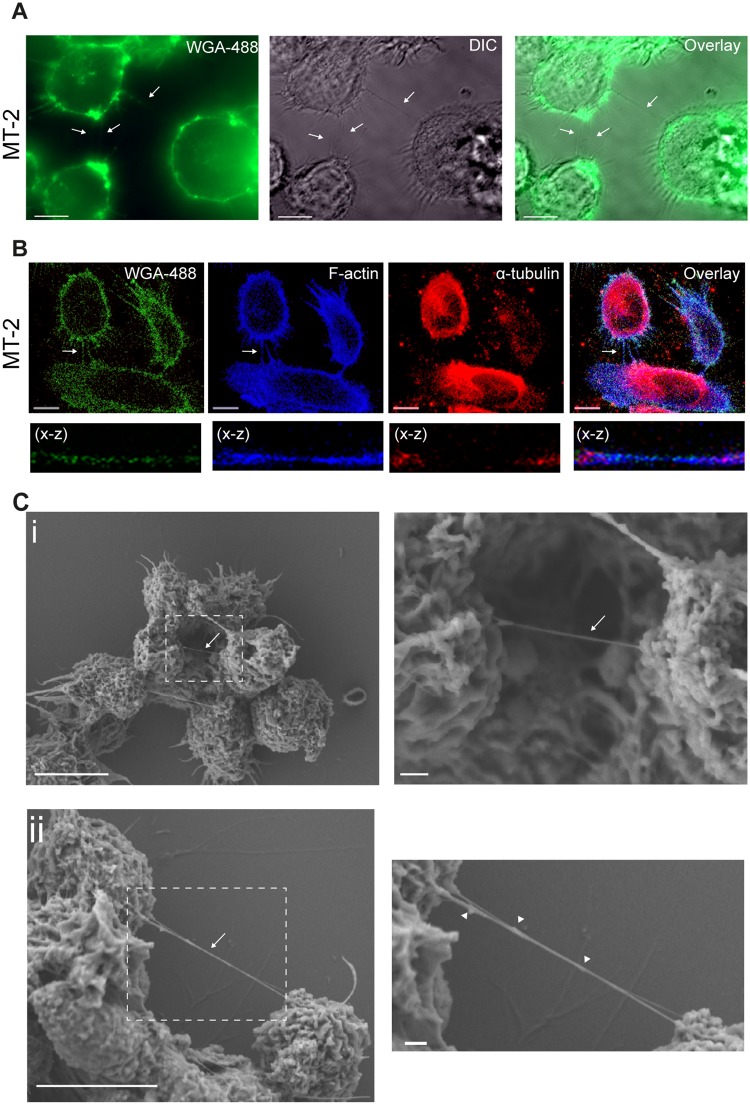
HTLV-1 expressing cells form tunneling nanotubes (TNTs). (**A**) MT-2 cells stained with WGA-488 (cellular membranes, green) and investigated by fluorescence microscopy. Arrows indicate thin membrane embedded intercellular connections. (**B**) MT-2 cells stained with WGA-488 (green), F-actin phalloidin-AF350 (blue) and an anti-α-tubulin antibody (red) and analyzed by confocal microscopy. Arrows indicate a TNT. The X-Z plane is obtained from a Z-stack (total 24 slices of 1 µm each). (**C**) Scanning electron microscopy (SEM) of MT-2 cells connected by TNTs. Zoomed images to the right. Arrows indicate TNTs, arrowheads indicate bulges in TNTs. Images were captured by scanning electron microscope Jeol JSM-7400F LEI 4.0 kV, x3000 (x3700 for D2) and WD 8.0 mm. Confocal microscopy images were acquired by LSM780 confocal microscope (Zeiss) and fluorescence microscopy was acquired by AxioObserver Z1 (Zeiss). All images are representatives of three independent experiments, except for (C) that was performed once. All scale bars 10 µm except zoomed SEM images; 1 µm. Adobe photoshop CS6 was used to prepare the images. The contrast was enhanced on the whole image to better represent and visualize the thin TNT structures.

We previously reported the presence of cellular conduits in HTLV-1 expressing cells^34^. At that time these structures did not fulfil the strict criteria of being a TNT and were consequently named cellular conduits^34^. Since HIV-1 has been shown to facilitate TNTs for viral spread between T-cells and macrophages^43,44,48^ we wanted to determine if HTLV-1 infected cells formed TNTs as well. The definition of a TNT in the present study is; a thin (200 nm in diameter, >5 µm length) membrane embedded, actin-containing, tubulin absent, structure interconnecting two cells simultaneously hovering above the surface of the well. Cytoplasmic bridges, the TNT-like structures following cell division, were excluded through the characteristic mid-body^48,49^. TNTs are very fragile structures *in vitro*, and thus, cellular fixation; in particular of suspension cells that are semi-adherent to fibronectin coated surfaces, will result in TNT breakage^50^. Hence, suspension cells are generally analysed by live cell microscopy. However, because MT-2 cells produce infectious HTLV-1 viral particles^51,52^, they require fixation before analysis by confocal microscopy in regular microscopy core facilities. Thin intercellular connections >5 µm were found in the MT-2 cells (Fig. 1A). These connections did not adhere to the plastic surface and contained F-actin, but no α-tubulin and therefore qualified, according to the TNT definition, as TNTs (Fig. 1B). To further analyse the TNTs in more detail, we performed scanning electron microscopy (SEM). In the MT-2 cells, TNTs hovering above the substratum were found interconnecting cells and interestingly, at one end only, a branched anchoring was frequently observed (Fig. 1C, arrows) as also described for other cellular systems^53^. Furthermore, knob-like structures were observed in the TNTs (Fig. 1C, ii, arrowheads) which could indicate transport of cargo through these structures as previously described^54^.

**Figure 2:**
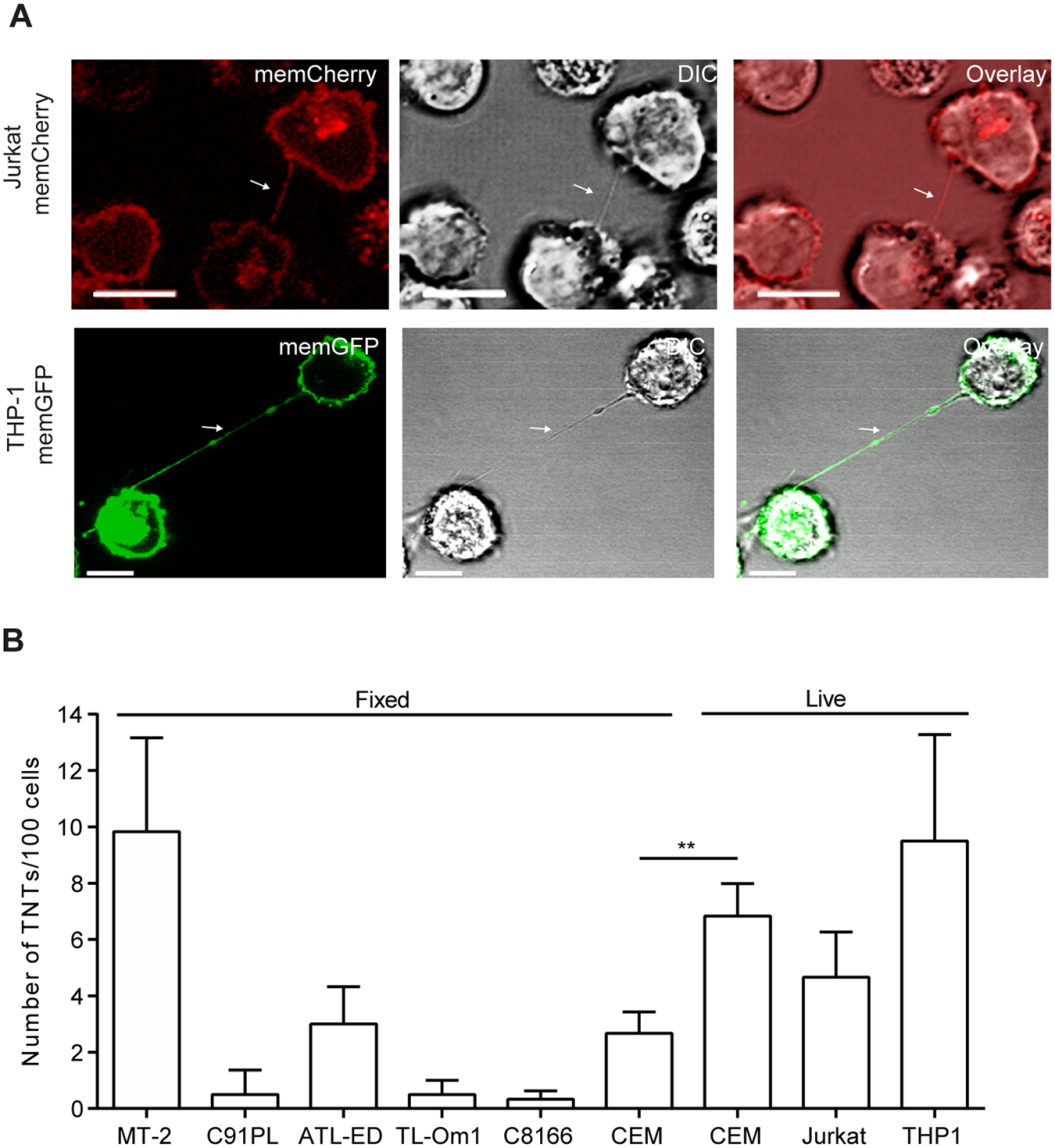
TNTs in HTLV-1 positive and negative cells. (**A**) Jurkat-memGFP (green) and THP-1-memCherry (red) cells analyzed by confocal microscopy. Arrows indicate TNTs. The images are representatives of three independent experiments. (**B**) TNTs were quantified in HTLV-1 transformed cells (MT-2, C91PL, ATL-ED, TL-Om-1 and C8166) and in HTLV-1-negative cell lines; T-cells (CEM and Jurkat) and monocytes (THP-1). TNT quantifications in HTLV-1-negative cells were performed by live cell microscopy where the Jurkat-memCherry and THP-1-memGFP cells were directly imaged while CEM cells were stained with WGA-488 prior to imaging. All HTLV-1 transformed cells were fixed and stained with WGA-488 before analyzed by microscopy. For the TNT quantification, 100 cells were counted in duplicate wells for the presence of TNTs connecting two cells as described earlier^49^. Results represent three independent experiments performed in duplicates and error bars mean ± SD. Unpaired t-test was performed to evaluate statistical significance. F-test was performed for individual variation. GraphPad Prism (Version 6.03) was used (**p < 0.01). Scale bars 10 µm. Adobe photoshop CS6 was used to prepare the images. The contrast was enhanced on the whole image to better represent and visualize the thin TNT structures.

The HTLV-1 infected cell lines C91PL (HTLV-1 producing), C8166 (no viral production, but expression of Tax protein), two ATL-derived T-cell lines TL-Om1 and ATL-ED (no viral production or Tax expression) and the HTLV-1-negative cell lines Jurkat (T-cells), CEM (T-cells) and THP-1 (monocytes) where further compared to MT-2 cells with respect to TNT forming capabilities. All HTLV-1 transformed cell lines were fixed and stained with WGA-Alexa488 prior to analysis by microscopy, whereas the HTLV-1-negative cell lines were analysed by live cell imaging. In order to visualize TNTs without the need of cellular staining, Jurkat and THP-1 cells were stably transduced with memGFP or memCherry, respectively, (see material and methods) to directly visualize TNTs and distinguish the originating cell (Fig. 2A). To compare TNT formation in the different cell lines, TNTs/100 cells were quantified as described earlier^49^. MT-2 cells consistently formed more TNTs when compared to the other HTLV-1 positive cells (Fig. 2B). TNT formation in MT-2 cells was similar to that quantified of unfixed THP-1 and CEM cells and since fixation will reduce the number of TNTs as stated above, and demonstrated by comparing fixation of CEM cells resulted in a 60.9% (p < 0.0065) reduction in detectable TNTs (Fig. 2B), it is likely that the TNT numbers in unfixed MT-2 cells will be higher.

### HTLV-1 expressing cells form TNTs with non-infected T-cells and monocytes containing Tax and Gag

**Figure 3:**
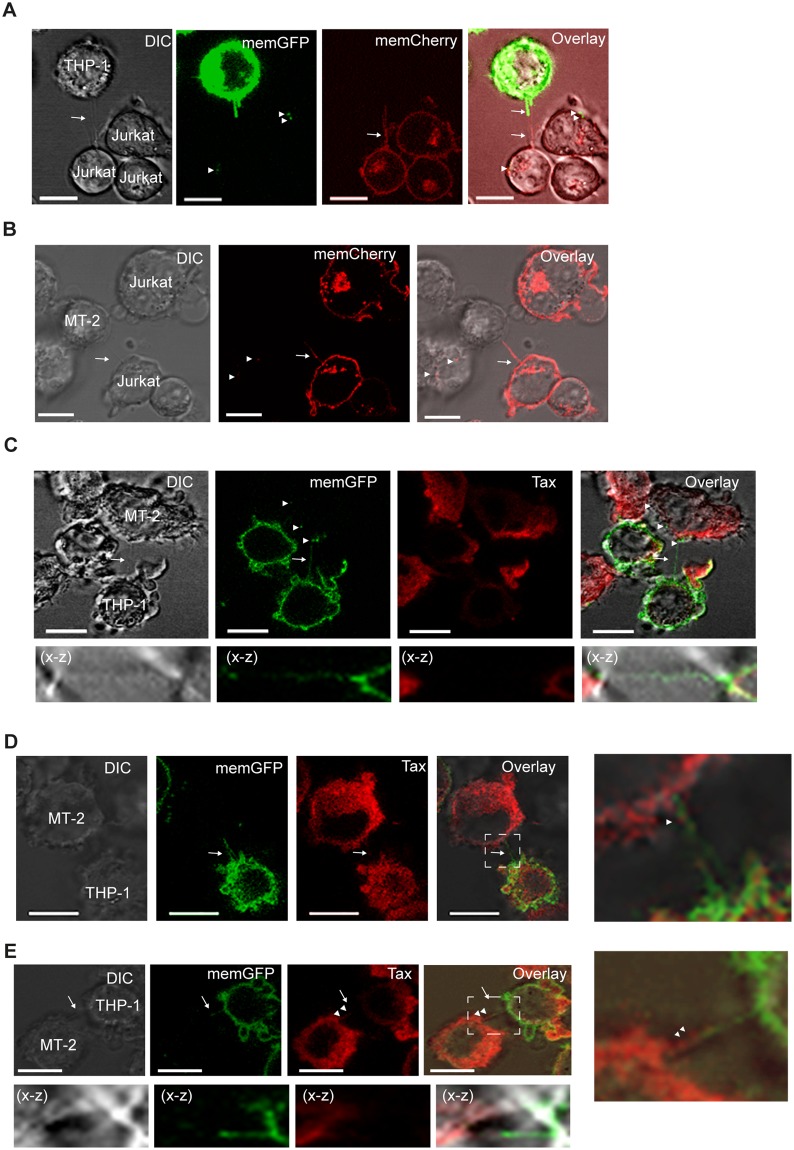
HTLV-1 expressing cells form TNTs with non-infected T cells and monocytes and contain Tax. (**A**) THP-1-memGFP and Jurkat-memCherry cells were co-cultured overnight and presence of TNTs was analyzed by DIC and fluorescent confocal microscopy. Arrows indicate TNTs and arrowheads memCherry and memGFP. (**B**) MT-2 (unstained) and Jurkat-memCherry cells were co-cultured overnight and the presence of TNTs was analyzed by DIC and fluorescent confocal microscopy. Arrows indicate TNTs and arrowheads memCherry. (**C**–**E**) MT-2 and THP-1-memGFP cells were co-cultured overnight and immunostained for Tax (red). The presence of TNTs was analyzed by DIC and fluorescent confocal microscopy. Arrows indicate TNTs and arrowheads memGFP (green) or Tax (red). All images are representatives of two independent experiments performed in duplicates. The X-Z plane is obtained from a Z-stack (total 32 slices of 1 µm each). Scale bar 10 µm. Adobe photoshop CS6 was used to prepare the images. The contrast was enhanced on the whole image to better represent and visualize the thin TNT structures.

To further investigate the TNT connections between HTLV-1 positive and HTLV-1 negative cells, MT-2 cells were co-cultured with Jurkat or THP-1 cells. These cultures were compared to co-cultures of uninfected Jurkat and THP-1 cells. Firstly, in cultures of THP-1 (memGFP) and Jurkat (memCherry) cells, we found that TNTs could be generated from both cells. TNTs that originated from Jurkat cells toward THP-1 cells and vice versa were detected (data not shown). In addition, TNTs were formed that originated from both cell types simultaneously (Fig. 3A, arrows) as seen in other systems^48^. Further, memGFP dots were observed in the Jurkat cells possibly indicating intercellular transport from the THP-1 cells (Fig. 3A, arrowheads). When unstained MT-2 cells were co-cultured with Jurkat (memCherry) or THP-1 (memGFP) cells, TNTs were readily formed between the HTLV-1 positive and negative cells (Fig. 3B,C, arrows). When the origin of TNTs connecting MT-2 and THP-1 cells were quantified we found that 48% of the TNTs originated from the MT-2 cells and 52% from the THP-1 cells (Supplementary Fig. 1A) As with uninfected cells, memCherry and memGFP dots were observed in the MT-2 cells. A 3D reconstruction was made of the cells from Fig. 3C and show memGFP as well as Tax present in recipient cells (Supplementary Video 1). This suggests that membrane exchange occurs from the HTLV-1-negative cells to MT-2 cells (Fig. 3B,C, arrowheads) possibly by TNTs or other mechanisms such as exosomes and phagocytosis. When the co-cultured MT-2 and THP-1 cells were immunostained for intracellular Tax protein, Tax was found localized to TNTs, and could be detected in TNTs originating from the THP-1 cells (Fig. 3D) and in TNTs generated from both MT-2 and THP-1 cells (Fig. 3E). The specificity of the immunostaining was verified by incubating mono-cultures of THP-1 (memGFP) cells with the anti-Tax antibody and in addition a Tax negative THP-1 cell is shown in a co-culture with MT-2 and THP-1 (memGFP) cells (Supplementary Fig. 1B,C). This demonstrated that HTLV-1 negative cells can generate TNTs to communicate with MT-2 cells with potential transport of membrane components and Tax protein.

**Figure 4:**
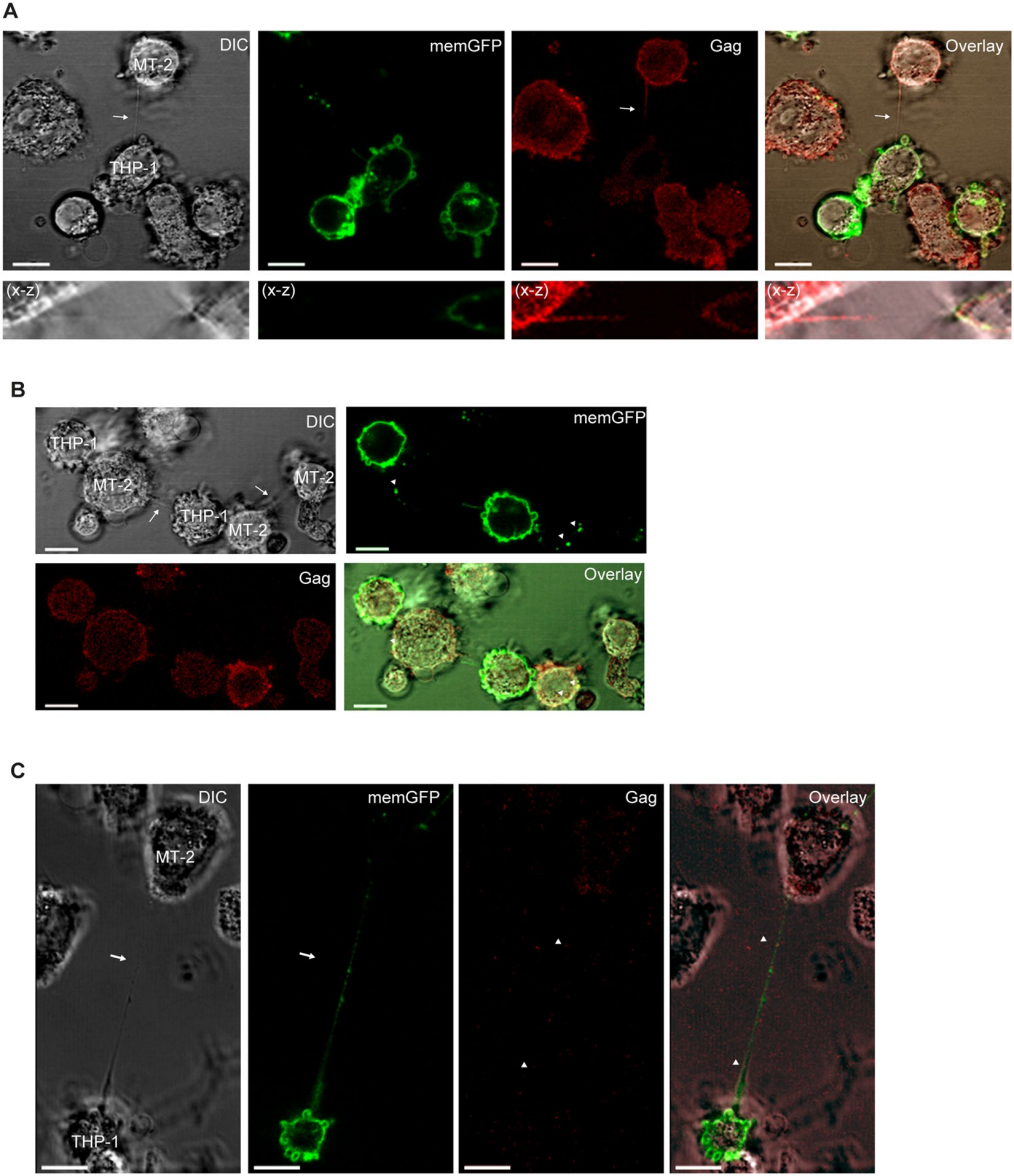
Gag present in TNT connecting HTLV-1 expressing cells and non-infected monocytes. (**A**–**C**) MT-2 cells and THP1**-**memGFP (green) cells were co-cultured overnight, fixed and stained for p24Gag (red) before investigated for TNT connections by DIC and fluorescent confocal microscopy. Arrows indicate TNTs and arrowheads Gag. The X-Z plane is obtained from a Z-stack (total 23 slices of 1 µm each). Images are representatives of three independent experiments performed in duplicates. Scale bar 10 µm.

Next, MT-2 cells co-cultured with THP-1 (memGFP) were immunostained for Gag. We found Gag proteins localized to the TNTs, generated by MT-2 cells towards THP-1 cells which could indicate transport of Gag from the MT-2 cells towards the THP-1 cells (Fig. 4A). When TNTs formed between MT-2 and THP-1 were quantified for the presence of Gag or Tax, we found an average of 40% of TNTs containing Gag and 18% containing Tax (Supplementary Fig. 1D). Both tight, direct cell-to-cell contacts and TNTs were observed between MT-2 and THP-1 cells where some cells also simultaneously engaged in TNT communication and direct contacts (Fig. 4A). We further observed that when a THP-1 cell and a MT-2 cell formed a tight connection both the THP-1 cell and the MT-2 cell formed TNT connections with two MT-2 cells on separate sites of the direct contact site (Fig. 4B). Finally, both Gag and memGFP proteins were also localized to the TNTs originating from THP-1 cells and connecting to MT-2 cells (Fig. 4C). This memGFP or Gag was demonstrated to be intracellular by a 3D reconstruction of a Z-stack from Fig. 4A (Supplementary Video 2).

### Cytarabine treatment reduces TNT numbers in HTLV-1 expressing cells

**Figure 5:**
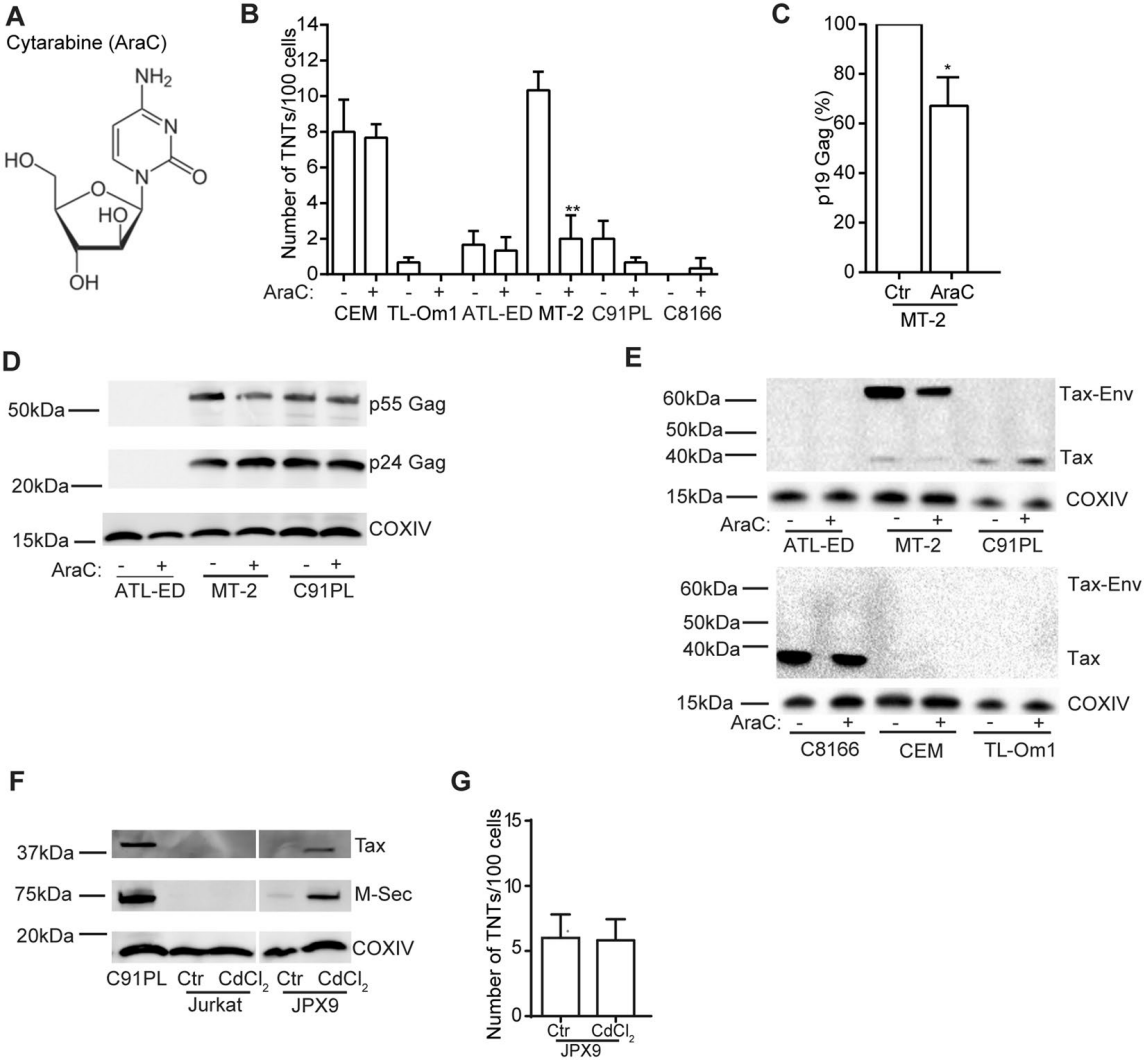
Cytarabine treatment reduces TNT numbers in HTLV-1 expressing cells. (**A**) Molecular structure of cytarabine (AraC). (**B**) Quantification of TNTs in cell lines before and after cytarabine (AraC) treatment (1 µM, 24 h). (**C**) Quantification of p19 Gag (pg/ml) by the HTLV-1 antigen capturing ELISA kit in MT-2 supernatant before and after treatment with AraC (1 µM, 24 h). (**D**) Immunoblotting of ATL-ED, MT-2 and C91PL cells before and after treatment with AraC (1 µM, 24 h) with antibodies against p24/p55 Gag and COXIV (loading control). (**E**) Immunoblotting of C8166, CEM, TL-Om1, ATL-ED, MT-2 and C91PL cells before and after treatment with AraC (1 µM, 24 h) with antibodies against Tax and COXIV (loading control). (**F**) Immunoblotting of Jurkat and JPX9 (Tax wt) cells, before and after CdCl_2_ treatment; antibodies against Tax, M-Sec and COXIV (loading control). Lysates of C91PL cells: positive control of Tax and M-Sec expression. Results are representative of three independent experiments. (**G**) Quantification of TNTs in JPX9 cells before and after treatment with CdCl_2_. All error bars indicate the mean ± standard deviation. For TNT quantifications; all data are presented as three independent experiments in duplicates, p19 Gag quantifications; all data are presented in percentage as three independent experiments. The immunoblots are representatives of three independent experiments. Unpaired t-test was performed to evaluate statistic significance. F-test was performed for individual variation. GraphPad Prism (Version 6.03) was used (*p < 0.05, **p < 0.01). Scale bars: 10 µm. Full-length immunoblots are presented in Supplementary Fig. 2.

Cytarabine (AraC) is a deoxycytidine analogue (Fig. 5A) which combined with an anthracycline represents standard induction therapy for treatment of AML patients^55^. We have previously demonstrated that clinical relevant doses of cytarabine^56^ treatment decreases the number of TNTs in AML cells involving the NF-κB pathway^49^. Since HTLV-1 cells, similar to AML cells are known to express a constitutive active NF-kB pathway mediated by Tax^57,58^, we studied the effect of cytarabine on TNTs in HTLV-1 infected cells and the HTLV-1 negative T-cell line CEM. MT-2, C91PL, TL-Om1, ATL-ED and CEM cells were treated with 1 µM cytarabine for 24 h followed by fixation of the HTLV-1 expressing cell lines, and TNT quantification compared to non-treated cells (Fig. 5B). This caused 11% cell death in the MT-2 cells and <5% in the other cell lines as determined by Hoechst 33342 staining following microscopy (data not shown) and TNT numbers were quantified between live cells only and these were significantly reduced in the MT-2 cells after cytarabine treatment, whereas no significant change was found in CEM cells (Fig. 5B). The other HTLV-1 positive cell lines that expressed low TNT numbers demonstrated minor reductions after cytarabine treatment. To determine if cytarabine treatment affected viral release, p19Gag (pg/ml) was measured in the supernatant of mock treated or 1 µM cytarabine treated MT-2 cells. Cytarabine treatment resulted in approximately 30% reduction in p19Gag (Fig. 5C). When intracellular p24Gag in MT-2 cells was evaluated by immunoblotting after 24 h of cytarabine (1 µM) treatment, we found a reduction in the p55Gag precursor and an increase in p24Gag (Fig. 5D). Whereas this was not as evident in the cytarabine treated C91PL cells (Fig. 5D). Further, cytarabine treatment also resulted in reduced Tax protein expression in MT-2 cells (Fig. 5E) indicating an association between Tax and TNT numbers.

To further investigate the role of Tax in TNT formation, we used the Jurkat derived cell line JPX9 which can be induced with CdCl_2_ to express Tax^59^. Although we could detect induction of Tax protein in the JPX9 cells by immunoblotting (Fig. 5F), we found no difference in TNT numbers (Fig. 5G). The induction of wildtype Tax also increased expression of the M-Sec (TNF alpha inducing protein 2) protein (Fig. 5G), previously reported to be a central protein for TNT formation^60^. Based on this we concluded that Tax and M-Sec most likely are not essential for TNT formation in HTLV-1 infected cells.

### Cytarabine reduces viral transmission

**Figure 6:**
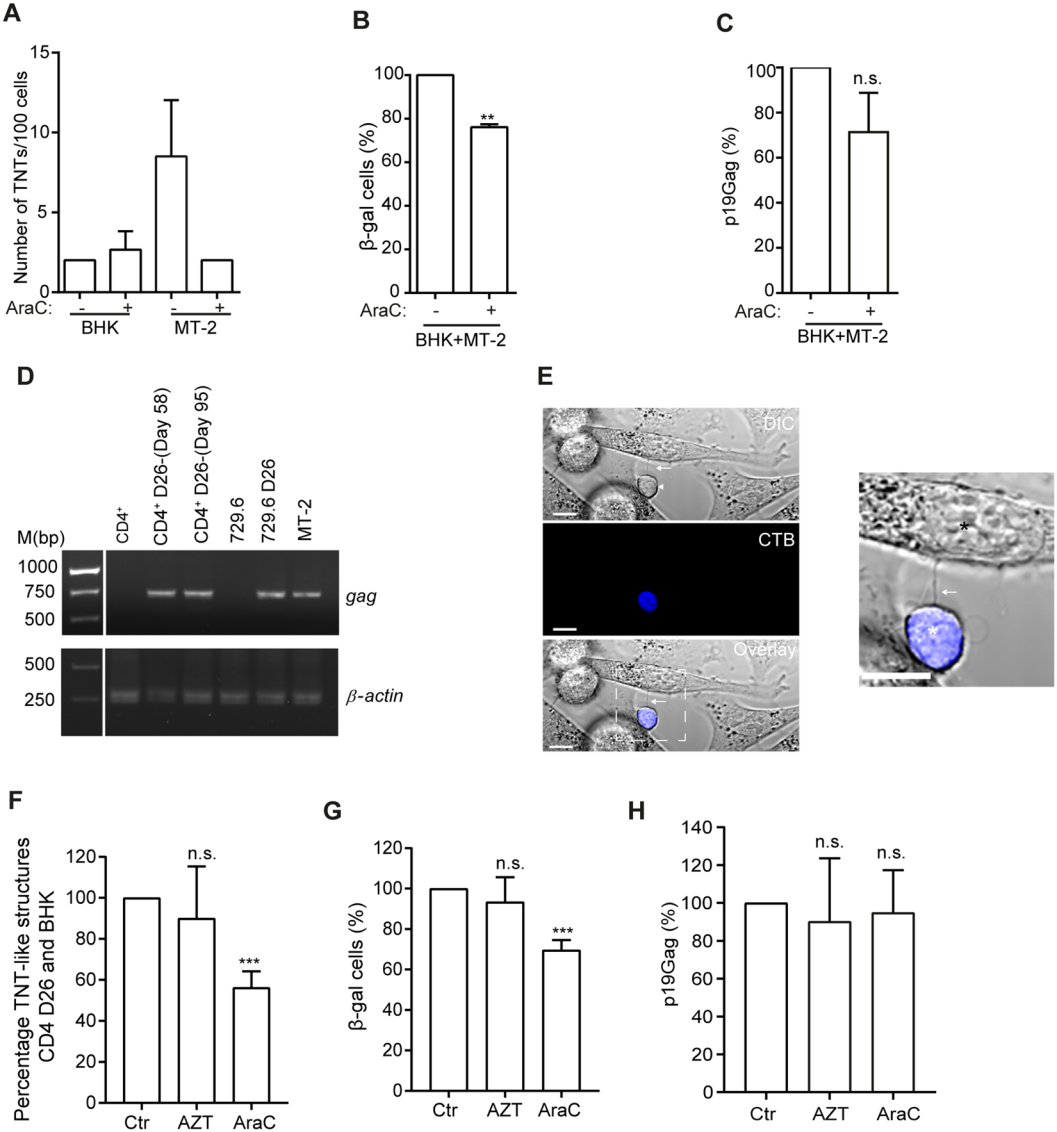
Cytarabine, but not AZT significantly inhibits viral transmission and TNT-like structures between primary CD4^+^-pAB-D26 and BHK1E6 cells (**A**) TNT quantification of BHK1E6 cells (BHK) and MT-2 cells before and after cytarabine (AraC) treatment (1 µM, 24 h). TNTs quantified between mono-cultures of BHK1E6 and MT-2 cells. (**B**) Co-culture of MT-2 and BHK1E6 (BHK) cells (24 h) with 1 µM AraC or ctr stained for β-galactosidase activity (blue cells), percentage of three independent experiments performed in duplicates. (**C**) Quantification of p19 Gag (pg/ml) in supernatants of co-culture experiments in (**B**) by the HTLV-1 antigen capturing ELISA kit (**D**) Validation of Gag presence in primary CD4^+^-pAB-D26 cells by PCR, uninfected CD4^+^ cells and 729.6 cells are shown as negative controls, 729.6-pAB-D26 and MT-2 cells are shown as positive controls. (**E**) TNT-like structure between cell tracker blue stained CD4^+^-pAB-D26 in co-culture with BHK1E6 cells, arrow indicate TNT-like structure. In zoomed image black star indicate BHK1E6 cell and white star CD4^+^-pAB-D26. Image is representative of three independent experiments performed in duplicates. (**F**) Quantification of TNT-like structures between CD4^+^-pAB-D26 and BHK1E6 cells co-cultured for 24 h with 10 µM AZT, 1 µM AraC or medium only. 100 CD4^+^ D26 cells were counted in each experiment. Mean percentage from three independent experiments performed in duplicates are shown. (**G**) Co-culture of CD4^+^-pAB-D26 cells and BHK1E6 cells (24 h) with ctr, 10 µM AZT or 1 µM AraC stained for β-galactosidase activity (blue cells), percentage of three independent experiments performed in duplicates. (**H**) Quantification of p19 Gag (pg/ml) in supernatants of co-culture experiments in (**G**) by the HTLV-1 antigen capturing ELISA kit. All p19 Gag quantifications are presented in percentage of three independent experiments. All error bars indicate the mean ± standard deviation. Unpaired t-test was performed to evaluate statistical significance. F-test was performed for individual variation. GraphPad Prism (Version 6.03) was used (**p ≤ 0.01, ***p ≤ 0.001, n.s. not significant). Full-length gel images are presented in Supplementary Fig. 4.

To determine if cytarabine affected cell-to-cell transfer and infection, we utilized the BHK1E6 cell reporter system. The BHK1E6 cells contains the *lacZ* gene driven by the HTLV-LTR promoter, allowing β-galactosidase to be used as a measure of LTR activation by Tax^61^. First, the BHK1E6 cells were investigated for the presence of TNTs before and after treatment with cytarabine (1 µM) for 24 h. The BHK1E6 cells expressed on average 2 TNTs/100 cells which did not change after cytarabine treatment and no cell death was found in the BHK1E6 cells after treatment as compared to MT-2 cells (Fig. 6A). TNT-like structures were observed interconnecting MT-2 and BHK1E6 cells after co-culture (data not shown) and the percent of β-galactosidase production was significantly reduced by approximately 25% in the presence of cytarabine (1 µM, 24 h) and a consistent, but not significant, reduction of cell supernatant p19Gag was found (Fig. 6B,C). To investigate viral transfer by the use of newly HTLV-1 infected cells, we infected primary sorted CD4^+^ cells from healthy peripheral blood mononuclear cells by co-culturing with lethally γ-irradiated 729.6 cells expressing the HTLV-1 molecular clone pAB, as previously described^37^. This molecular clone contains an *orf-I* encoding for an aspartic acid in position 26 resulting in equal expression of p12 and p8 proteins^36^ and therefore these newly infected CD4^+^ cells are named CD4^+^-pAB-D26. Cell purity of the CD4^+^-pAB-D26 cells was measured by flow cytometry while verification of the presence of HTLV-1 *Gag* was performed by PCR on isolated DNA and the proviral load was found to be 265.6% at day 58 in culture (Fig. 6D and Supplementary Fig. 3). The calculation of proviral load (%) was based on the copy number of *HTLV-1* per 100 of copy number of the *RNase* P gene. When the CD4^+^-pAB-D26 cells were co-cultured with the BHK1E6 cells TNT-like structures were observed between these cells (Fig. 6E). Following treatment for 24 h with cytarabine (1 µM, 5% cell death measured by Hoechst 33342 staining) or the reverse transcriptase inhibitor AZT (azidothymidine, zidovudine, 10 µM, 4% cell death measured by Hoechst 33342 staining), included as one of the treatment options of ATL patients today^62^, we found that the number of TNT-like structures connecting the CD4^+^-pAB-D26 cells and BHK1E6 cells were reduced by 44% after treatment with cytarabine, but not AZT (Fig. 6F) and interestingly, cytarabine treatment resulted in a 30% decrease in number of β-galactosidase positive cells (Fig. 6G). No significant change of p19Gag in the supernatant of the co-culture was found (Fig. 6H), therefore not explaining the reduction of blue cells (β-gal positive) in the transmission assay. We have previously shown that cytarabine can inhibit TNT formation^49^, but here we do not exclude other transmission mechanisms of Tax transfer to the BHK1E6 cells. Since Tax have been found in exosomes^63^ the difference in reduction between TNT-like structures and β-galactosidase positive cells could be explained by an exchange of vesicles or other means of Tax transfer. In conclusion we find that 24 h treatment of cytarabine reduce viral transmission, not found after AZT treatment.

### p8 induces TNT formation in uninfected T-cells

**Figure 7:**
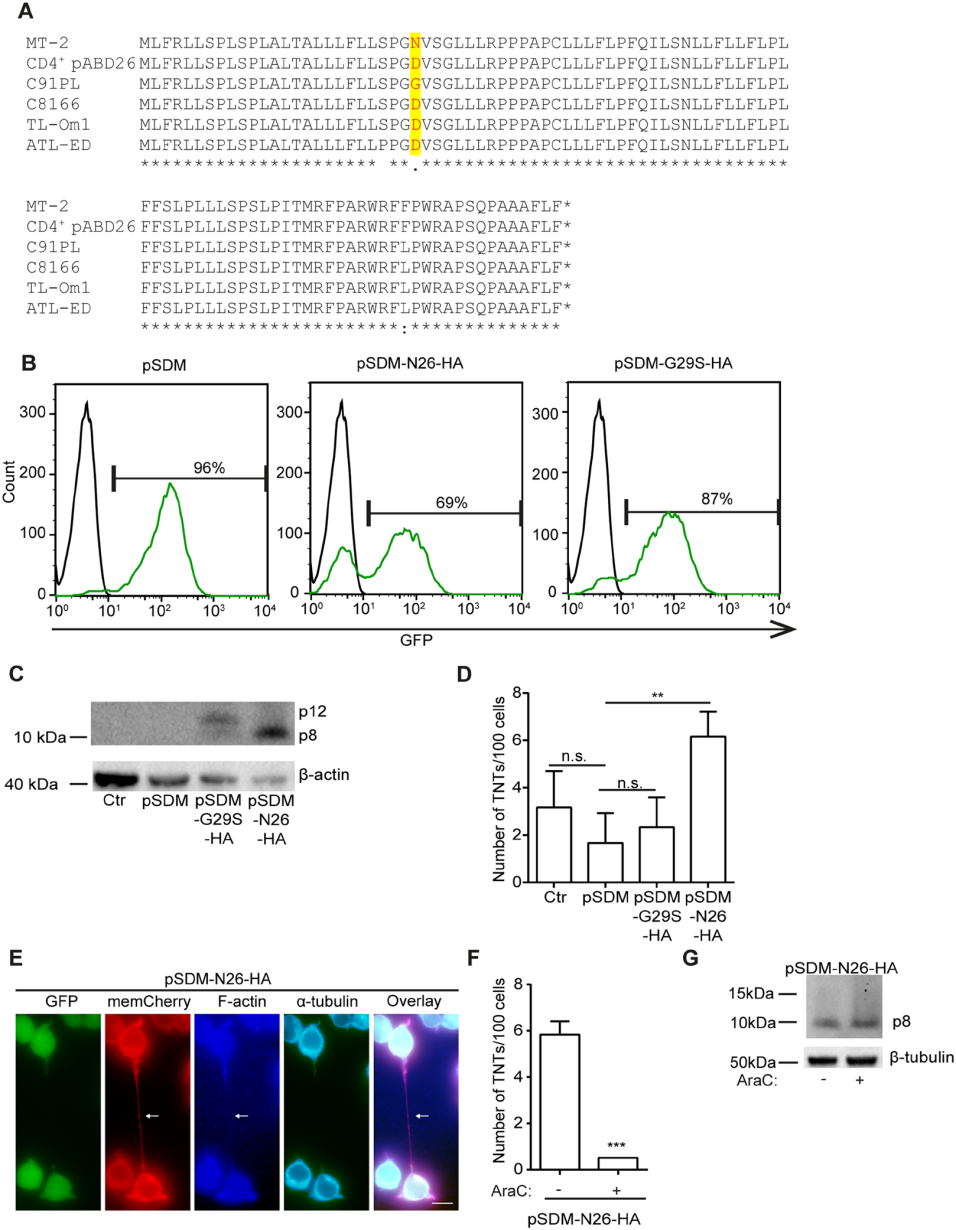
The p8 protein increases TNT formation. (**A**) Orf-I amino acid sequences of HTLV-1 positive cell lines. (**B**) Transduction efficiency determined by flow cytometry of percentage GFP positive cells in Jurkat-memCherry cells transduced with pSDM, pSDM-N26(p8)-HA or pSDM-G29S(p12)-HA. (**C**) Immunoblotting of Jurkat-memCherry cells (ctr), Jurkat-memCherry; pSDM, pSDM-G29S-HA and pSDM-N26-HA incubated with anti-HA antibody. β-actin was used as loading control. (**D**) TNT quantification of Jurkat-memCherry; pSDM, pSDM-G29S-HA and pSDM-N26-HA. Results are presented as mean from three independent experiments performed in duplicates. (**E**) TNT verification in Jurkat-memCherry-pSDM-N26-HA cells. The cells were fixed and stained with AF350 phalloidin to visualize F-actin (blue) and anti-α-tubulin to visualize tubulin (cyan, AF633), transduced cells are GFP positive, plasma membrane memCherry (red). Scale bar: 10 µm. Result is representative of three independent experiments. (**F**) Jurkat-memCherry-pSDM-N26-HA cells were untreated or treated with 1 µM cytarabine (AraC) for 24 h and TNTs were quantified in live cells. Results are shown as mean from three independent experiments performed in duplicates. (**G**) Immunoblotting of Jurkat-memCherry-pSDM-N26-HA cells not treated or treated with 1 µM AraC incubated with anti-HA antibody. β-tubulin was used as loading control. Representative blot of three independent experiments is shown. Unpaired t-test was performed to evaluate statistic significance. F-test was performed for individual variation. GraphPad Prism (Version 6.03) was used (**p < 0.01, ***p ≤ 0.001). Full-length immunoblots are presented in Supplementary Fig. 5. Adobe photoshop CS6 was used to prepare the images. The contrast was enhanced on the whole image to better represent and visualize the thin TNT structures.

Because we earlier reported that p8 induced cellular conduits and we observed a difference in TNT forming capabilities among the HTLV-1 positive cell lines, we investigated the *orf-I* sequence in these cells (Fig. 7A). Interestingly, the MT-2 cell line which had the highest TNT numbers, contained a provirus encoding an asparagine at position 26 (N26) of *orf-I*, an isoform we previously demonstrated to mainly express the p8 protein an inducer of viral transmission^34,36^. Except for the C91PL cells, the remaining HTLV-1 cell lines, including the primary infected CD4^+^ pAB-D26 cells, encoded the isoform with an aspartic acid at position 26 (D26) resulting in approximately equivalent expression of p12 and p8^36^. The C91PL cell line contained provirus encoding a glycine in position 26 (G26) also found to express equal amounts of p12 and p8 proteins (unpublished data).

To further investigate the role of p8 in TNT formation, we stably transduced Jurkat memCherry cells with either a GFP-expressing lentiviral empty vector control (pSDM) or pSDM expressing an orf-I unable to be proteolytically cleaved and therefore producing p12 only; G29S^33^ (p12)-HA or N26 (p8)-HA producing mainly the p8 protein^36^. The transduction efficiency was determined by flow cytometry as percentage GFP-positive cells; pSDM: 96%, pSDM-N26(p8)-HA: 69% and pSDM-G29S(p12)-HA: 87% (Fig. 7B) and expression was verified by immunoblotting (Fig. 7C). Following this, live cell TNT quantification was performed including GFP-positive cells only. We found that compared to the Jurkat-memCherry cells only, the transduction by pSDM resulted in reduced TNT numbers, however, no significant differences were found between the pSDM cells and the pSDM-G29S(p12)-HA cells (Fig. 7D). The pSDM-N26(p8)-HA positive cells showed a significant induction of TNTs (*p* = 0.0088) compared to the pSDM or pSDM-G29S(p12)-HA positive cells (Fig. 7D). The structures were verified as containing F-actin and devoid of tubulin. However, tubulin could be detected at both ends of the TNT structure (Fig. 7E), as also observed by others^64^. Consistent with what we observed in HTLV-1 producing cells, treatment of pSDM-N26(p8)-HA expressing cells with 1 µM cytarabine (8% cell death measured by Hoechst 33342 staining) resulted in a significant (p < 0.0001) down-regulation of the TNT formation (Fig. 7F) without affecting p8 expression (Fig. 7G).

Taken together, we have shown that HTLV-1 expressing cells are interconnected by the strictly defined intercellular structures TNTs as well as with uninfected T-cells and monocytes. TNTs were found to originate from either HTLV-1 positive cells or HTLV-1 negative cells. Using memGFP and memCherry stably expressing cells we found the presence of both memGFP, memCherry, Tax and Gag proteins in the TNTs indicating potential transport of these proteins through the TNTs. Cytarabine treatment resulted in a reduction of TNTs, virus production and viral transmission. Expression of orf-I-N26 in Jurkat cells enhanced number of TNT formation significantly reduced by cytarabine treatment.

## Discussion

TNTs represent direct long distance cell-to-cell communication where cells can exchange material. TNTs have been suggested to play a role in different diseases such as cancer and infectious diseases involving chemoresistance and spread of pathogens^47–49,65–70^.

Retroviruses exploit host cells for their replication and viral spread. HTLV-1, in particular, is highly dependent on cell-to-cell interaction between immune cells for successful transfer and establishment of productive infection^28^. Here we find that the HTLV-1 infected cells fulfilled the strict definition for being a TNT. These cells expressed thin intercellular connections at least 5 µm in length that lacked contact with the substratum. MT-2 cells contained F-actin and lacked tubulin and by scanning electron microscopy (SEM) these structures were verified to be thin (<200 nm) and interestingly often with a branched anchor point, which has been previously described for TNTs^53^. These TNTs were frequently observed having multiple knobs indicating transport.

Variation in the number of TNTs was seen among the HTLV-1 infected cell lines. The ATL T-cell lines (TL-Om1, ATL-ED) which have integrated provirus, but do not express Tax or release virus, formed less TNTs than the HTLV-1 producing cell line MT-2 and showed more similarity to the HTLV-1 expressing cell line C91PL and the C8166 cell line, containing a defect provirus, but expressing Tax protein. However, TNT quantification was challenging in the C91PL and C8166 cells due to their cell culture growth in large clusters particularly difficult to separate into single cells. In cell cultures, many T-cell lines grow in clusters most likely reflecting expression of cell surface adhesion molecules and this expression and distribution could impact TNT formation. In cells that grow in such close proximity viruses will most likely exploit close contact cell-to-cell communication rather than long-distance TNT communication.

Both membrane nanotubes as well as cellular conduits have previously been reported to connect infected and uninfected T-cells^34,48^. Sowinski and co-workers demonstrated transfer of the Human Immunodeficency Virus type 1 (HIV-1) viral proteins Gag and Env between two populations of T-cells^48^. We showed previously that cellular conduits could be used to transport Gag and the HTLV-1 encoded p8 protein from MT-2 cells to uninfected Jurkat cells^34^. In addition to defining the cellular structures, we wanted to investigate, using co-culture systems, the cellular origin of the TNTs formed. For this purpose we generated Jurkat and THP-1 cells stably expressing memCherry or memGFP, respectively. Membrane nanotubes have previously been reported for Jurkat and THP-1 cells^48^. We were particularly interested in looking at TNT formation between T-cells and monocytes since we recently demonstrated viral DNA in three different monocyte subsets from HTLV-1 infected individuals^12^. In addition, HTLV-1 infection of the monocytes is associated with changes in surface marker expression and function^12^. First, we established that THP-1 and Jurkat cells could form TNTs with infected T-cells and these originated from both cell types. This suggested, similar to what was reported by Sowinski and co-workers for T-cells, that the TNTs formed between T-cells and monocytes are not open-ended^48^. The consequence of this feature is that potential transport of cargo is not passive solely through a sheared membrane but mostly involve an active and motor driven transport. Further verification of the components of the TNTs formed is needed to conclude how this transport is occurring, by staining with motor proteins such as Myosin Va^39,48,71^. We found that TNT formation between infected and uninfected cells originated from both; from MT-2 cells to Jurkat and THP-1 cells or from Jurkat and THP-1 cells to MT-2 cells. Interestingly, following quantification, the Tax protein was localized to 18% of TNTs connecting MT-2 and THP-1 cells. Tax protein was also found localized to TNTs originating from THP-1 towards MT-2 cells. This could indicate transport of Tax protein from MT-2 cells towards uninfected cells using TNTs generated by the uninfected cells. Since these HTLV-1 producing cells required fixation, we were unfortunately not able to perform time-lapse live-cell imaging to further investigate Tax transport through the TNTs. Because Tax protein has not been demonstrated to be part of the viral particle, transfer of Tax protein is likely independent of viral infection of the recipient cell. Tax transfer could induce a variety of changes in an uninfected cell as Tax has been shown to affect a large number of cellular pathways^58^. Future studies establishing assays to investigate functional effect of Tax transfer are needed. In addition, we observed GFP positive dots in the MT-2 cells, implying an exchange of cargo in both directions. These observations support the possibility that HTLV-1 infected cells can utilize TNTs generated from an uninfected cell towards the infected to transport viral proteins, signaling molecules and the virus itself. Consistent with this, we found Gag in 40% of the TNTs connecting MT-2 cells and THP-1 cells. As seen with Tax, the Gag protein was also observed in the TNTs originating from the THP-1 cells. The Gag protein in the TNTs could represent Gag included as part of the viral particle, and/or it can also represent the Gag protein alone. Future studies are needed to better characterize virus particles actively transported through the TNTs.

We frequently observed that TNTs formed from one or both cells already in direct contact with other cells. This could point to that the previously described virological synapse and biofilm-like structures could be processes occurring simultaneously with TNT formation and one process could also activate the start of another. Meaning that when TNTs are used for transfer of virus and viral proteins it could be part of several approaches each cell can exploit for viral spread.

We have previously shown that TNT formation was downregulated by cytarabine treatment through a NF-кB dependent mechanism in AML cells^49^. Since HTLV-1 infected cells share some features with AML cells, such as constitutive activation of the NF-кB-pathway, MT-2 cells were treated with a clinically relevant^56^ and pre-apoptotic dose of cytarabine (1 µM) for 24 h (≤11% death). This treatment resulted in reduced TNT numbers and reduced levels of Tax protein. Interestingly, in MT-2 cells we found that, the p24 Gag protein was slightly increased after cytarabine treatment with a corresponding decrease in the precursor p55 Gag. We also noted a 33% reduction in the amount of supernatant p19 Gag. This suggest that cytarabine could alter Gag processing and the budding process of the virus. Since Tax is known to cause constitutive activation of NF-κB and cytarabine decreased Tax protein levels, we investigated the potential role of Tax in TNT formation using a Tax inducible Jurkat-derived cell line^58,72^. Induction of Tax showed an increase of the tumor necrosis factor α inducible protein 2 (TNFαIP2) also called M-Sec. M-Sec is suggested to be a key player in TNT formation^44,60^. However, no significant differences in TNT numbers was found between the Tax-induced cells and non-induced cells, consistent with our previous results^34^. This indicates that in these T-cells TNT formation is not significantly regulated by M-Sec and/or Tax expression and that TNT formation most likely is regulated different in AML cells compared to HTLV-1 infected cells.

We demonstrate in this study that the viral protein p8 induced TNT formation and that the variation found among the HTLV-1 positive cell lines corresponded with the *orf-I* isoform expressed. The cell lines which had low TNT formation (TL-Om1, ATL-ED, C91PL and C8166) all encoded an *orf-I* isoform previously demonstrated to express both p12 and p8 proteins; whereas MT-2 cells encoded for an *orf-I* isoform mostly expressing the p8 isoform. Likewise, in T-cells transduced with lentivirus that expressed *orf-I*-N26, thus p8, we saw an increase in TNT formation which was down-regulated by cytarabine treatment.

To investigate if HTLV-1 transfer was affected by cytarabine, we performed a viral transmission assay by co-culturing BHK1E6 containing a lacZ reporter gene driven by the HTLV-1 LTR promoter with MT-2 cells. MT-2 cells generated TNT-like structures with the BHK1E6 cells and cytarabine treatment reduced the number of β-galactosidase positive BHK1E6 cells by 25% indicating that cytarabine can inhibit viral transfer.

The anti-viral effects of cytarabine and down-regulation of Tax make cytarabine an interesting drug for further studies. The Tax protein has been shown to have pleiotropic effects and is proposed to be involved in the transformation of T-cells in ATL^58,73^. Despite little or no viral expression about 40% of ATL patients continue to express Tax in their HTLV-1 infected T-cells^74^. Drugs that affect Tax expression and NF-κB activation have been used as therapeutics in ATL^58^. Others have shown that the combination of arsenic trioxide and interferon alpha is an effective ATL treatment due to degradation of Tax^75,76^. Cytarabine could therefore potentially represent an alternative for use in ATL treatment, further supported by the fact that the down-regulation of viral expression was caused by the use of a clinical relevant dose of cytarabine^56^. Since higher viral loads are associated with disease progression^77,78^ cytarabine, because of its effects on TNT formation and HTLV-1 transmission could represent a promising therapeutic. It would be of interest to further investigate the efficacy of cytarabine in *in vivo* animal models of HTLV-1.

## Material and Methods

### Cells and culture conditions

The HTLV-1 positive cell lines MT-2, C91PL, C8166, TL-Om1 and ATL-ED and the HTLV-1 negative cell lines Jurkat, THP-1 and the Tax inducible cells JPX9 cells (kind gift from the laboratory of Dr. Giam at USHS) were all grown in RPMI-1640 medium. The BHK1E6 (HTLV-1-LTR-lacZ) cells^61^ were grown in Dulbecco’s modified eagles medium (DMEM). All cell culture media were supplemented with 10% fetal bovine serum (FBS), 2 mg/mL L-glutamine, 100 U Penicillin and 100 µg/mL Streptomycin (Quality Biological). Human peripheral blood T-cells (or buffy coats/leukapheresis packs) were obtained from healthy donors on NCI IRB-approved NIH protocol 99-CC-0168. Research blood donors provided written informed consent and blood samples were de-identified prior to distribution, in accordance with the Declaration of Helsinki.

The primary CD4^+^-pAB-D26 cells were cultured in RPMI-1640 medium supplemented with 20% FBS and 100 U of interleukin-2 in addition to L-glutamine and antibiotics. The THP1-memGFP and Jurkat-memCherry cells were generated by transducing THP-1 and Jurkat cells with ready-to-use lentiviral particles expressing twenty amino acids of the N-terminal part of Neuromodulin containing a palmitoylation signal fused to GFP or Cherry (Takara, rLV-EF1-AcGFP-Mem-9; rV2.1A1.1941 C2, rLV.EF1.mCherry-Mem-9;rV2.1A1.2406 C2), respectively, according to manufacturer’s instructions. The THP1-memGFP and Jurkat-memCherry cells were sorted by BD FACS Aria SORP at the Flow Cytometry Core Facility, Department of Clinical Science, University of Bergen, Norway.

### TNT definition and quantification

70 000 cells were seeded on fibronectin (10 µg/ml) coated µ-wells (IBIDI) and incubated for 24 h without or with 1 µM cytarabine (AraC) (Hospira, 100 mg/ml). Cells were stained with 1.67 µg/ml wheat germ agglutinin (WGA) conjugated to Alexa Fluor 488 or 594 (Life Technologies) for 8 min at 37 °C and washed once with DPBS1x (Gibco) before fixation with 4% PFA (Electron Microscopy Sciences) and 0.2% glutaraldehyde (Electron Microscopy Sciences) for 15 min at room temperature (RT) and gently washed twice before examined by microscopy. The TNT in the present study is defined and identified as a thin structure (<200 nm) interconnecting two cells, without contact with the substrate. Further, these TNTs are verified by content of F-actin and lack of microtubules. 100 cells were counted as explained previously^49^ in each well in duplicates and each experiment was performed as independent triplicates, unless otherwise noted. Cell viability was monitored by Hoechst 33342 (Sigma) staining as described earlier^79^.

### TNT verification

Cells were stained with WGA-488 (1.67 µg/ml) for 10 min at RT, washed once with PBS, permeabilized with 0.2% TWEEN 20 (polyoxyethylene sorbitan monolaurate) (Bio-Rad) in PBS for 1–2 min, washed with PBS and incubated in 0.5% BSA in PBS for 1 h at RT. Following this, the cells were stained for F-actin by incubation with Alexa Fluor 350 phalloidin (0.33 µM) for 1 h at RT, washed once in PBS and stained for tubulin by incubation with an α-tubulin antibody (1:100, Sigma) overnight at 4 °C. The cells were washed twice with PBS and incubated for 1 h at RT in the dark with goat anti-mouse Alexa Fluor 568 (1:5000, Invitrogen) or Alexa Fluor 633 (1:5000, Invitrogen). Finally, the cells were washed twice with PBS before analyzed by fluorescence microscopy.

### Immunofluorescence

Cells were fixed with 4% PFA (Electron Microscopy Sciences,) and 0.2% glutaraldehyde for 15 min, washed once with PBS before permeabilized with 0.2% TWEEN 20 in PBS for 1 min. Then the cells were washed once before blocked with 0.5% BSA in PBS for at 15 min at RT. The cells were then incubated for 1 h at RT with anti-p24 Gag antibody (ABL#4310 ABL inc., 1:50) or overnight at 4 °C with anti-Tax monoclonal antibody (Tab172^80^, 1:10), before washed twice with PBS followed by incubation with goat anti-mouse Alexa Fluor 568 as described earlier. Finally, the cells were washed twice with PBS before examined by fluorescence microscopy.

### Scanning electron microscopy (SEM)

Coverslips added to the wells of a 24-well plate were pre-coated with fibronectin (10 µg/ml) before seeding of 450 000 MT-2 cells per well. The cells were incubated for 24 h at 37°C before fixed overnight at 4°C using 2% glutaraldehyde in 0.1 M Na-cacodylate buffer. Cells were then washed 3 × 15 min with 0.1 M Na-cacodylate buffer, post-fixed in 1% osmiumtetraoksyd (OsO_4_) in Na-cacodylate buffer for 1 h at 4 °C, and washed 2 × 10 min in 0.1 M Na-cacodylate buffer before dehydration. Dehydration was performed as follows: 30% ethanol for 15 min, 50% ethanol for 15 min, 70% ethanol overnight, 96% ethanol for 20 min and 2 × 100% ethanol for 20 min. Following fixation and dehydration the coverslips were placed on SEM stubs before incubated in a heat-incubator overnight. Critical point drying was performed before the SEM stubs were coated with 5–10 nm gold/palladium before cells were investigated by scanning electron microscopy.

### JPX9 cells

JPX9 cells are stable transfected Jurkat cells containing wildtype Tax, inducible by a metallothionein promoter^59^. This promoter is activated by treatment with CdCl_2_. JPX9 cells (1.5–2.5 × 10^6^) were seeded in 6-well plates without or with 20 µM CdCl_2_ (Sigma) for 48 h and Tax expression was verified by immunoblot analysis. For microscopy analysis, 35 000 cells were seeded onto fibronectin pre-coated µ-wells, non-treated or treated with 20 µM CdCl_2_ for 48 h before stained with WGA-488 and examined by live cell microscopy.

### Co-culture assays

1 × 10^6^ BHK1E6 cells were seeded per well in 6-well plates and incubated overnight. The MT-2 cells were washed once with saline before co-culturing of 1 × 10^6^ cells with pre-seeded BHK1E6 cells. For primary CD4^+^-pAB-D26 cells the cells were centrifuged 5 min 1500 RPM and added RPMI-1640 medium supplemented with 20% FBS and 100 U of interleukin-2 in a 1 × 10^6^ cells/ml dilution and added to BHK1E6 cells. Co-cultured cells were untreated or treated with 1 µM cytarabine for 24 h. Supernatants were collected for analysis by the p19 Gag antigen capturing assay and the cells were washed twice with PBS, fixed in 4% PFA for 20 min and stained with the β-galactosidase kit (cat#: 35001, Active motif), according to manufactures protocol and incubated overnight at 37 °C. Following this the cells were washed twice with PBS blue cells were scored using light microscopy. TNT quantification of BHK1E6 cells in co-culture with MT-2 cells; 5000 BHK1E6 cells were seeded on fibronectin pre-coated µ-wells and incubated overnight before addition of 20 000 MT-2 cells without or with 1 µM cytarabine or 10 µM AZT (azidothymidine, zidovudine) treatment for 24 h. All experiments performed in duplicates and independently repeated three times. TNT quantification of BHK1E6 or MT-2 cells; 10 000 BHK1E6 cells or 70 000 MT-2 cells were seeded in separate wells without or with treatment with 1 µM cytarabine. Cells were fixed with 4% PFA for 20 min before examined by microscopy.

For co-culture of BHK1E6 and primary CD4^+^-pAB-D26 cells, 5 000 BHK1E6 cells were seeded on fibronectin pre-coated µ-wells (IBIDI) one day before addition of CD4^+^ pAB-D26 cells. CD4^+^-pAB-D26 cells were stained with celltracker blue CMAC (7-amino-4-chloromethylcoumarin, 25 µM) as described earlier^49^. 20 000 celltracker blue stained CD4^+^ pAB-D26 cells were added to the BHK1E6 cells and culture medium suitable for the CD4^+^-pAB-D26 cells was used for the co-culture added 1 µM cytarabine or 10 µM AZT. Cells were fixed and stained as described with MT-2 cells after 24 h co-culture.

For co-cultures between MT-2 and THP-1-memGFP cells, 56 000 MT-2 cells and 14 000 THP1-memGFP cells were plated on a fibronectin pre-coated 8 µ-well IBIDI. All experiments were independently repeated three times.

### p19 Gag antigen-capturing assay

Briefly, supernatants from cells were collected and centrifuged at 1800 rpm for 5 min at RT. The supernatants were transferred to a fresh collection tube and stored at −80°C until further analysis. The p19 Gag was measured by the p19 Gag antigen-capturing ELISA assay following the manufacturer’s instructions (ZeptoMetrix, Buffalo, NY).

### Immunoblotting

Cells were lysed in RIPA buffer for 10 min on ice before centrifugation 15 min 12000 RPM at 4°C and 40 µg protein was typically loaded on the SDS tris-glycine gels. Electrophoresis was performed by 150 volt for 1 h and wet blotting 1 h at 100 volt or by semi-dry blotting for 7 min, mixed range (Thermo-fisher). Membranes were blocked by 5 % BSA TBS-TWEEN for 1 h and incubated with primary antibodies at 4 °C overnight before washed with TBS-TWEEN, incubated with secondary antibody hrp-goat-anti-mouse/rabbit (1:1000) for 1 h at RT. Then membranes were washed two times with TBS-TWEEN and one time with TBS before developed by pico/pico plus/femto chemiluminescent substrate (Thermo Fisher) and developed digitally by the use of digital developer ImageQuant LAS 4000 (GE Healthcare Life Sciences) or ChemidocTM MP Imaging system (Bio-Rad) using Image Lab 5.0 (Bio-Rad, 2013) for image analysis. Antibodies for immunblotting: Tax (Tab172, 1:100), HTLV-1 p24Gag (ABL inc., 1:1000), COXIV (Abcam, 1:2500), anti-HA (Cell Signaling Technologies, 1:1000), β-actin (Abcam, 1:1000), β-tubulin (Abcam, 1:500).

### *Orf-I* sequencing

*Orf-I* sequencing was performed as described previously^36^ with slight modifications.

Approximately 1 × 10^6^ cells were collected and DNA was extracted using QIAamp DNA Blood Mini Kit (Qiagen), according to manufacturer’s instructions. DNA fragment covering *orf-I* was amplified by PCR using Platinum PCR SuperMix (Invitrogen) with primers (p12-F: 5′-CACCTCGCCTTCCAACTG-3′, p30-R: 5′-GGAGTATTTGCGCATGGCC-3′) and 300 ng of genomic DNA. The PCR reaction was carried out using an Eppendorf Master Cycler gradient PCR machine, for 35 cycles of; 94°C for 30 sec, 55°C for 30 sec, and 68°C for 50 sec. The PCR product was purified using Qiaquick PCR purification kit (Qiagen) and 50 ng DNA together with 0.64 picomol of primer (5′-CTGGACAGGTGGCCAGTA-3′) was used for sequencing. The Sanger sequencing was carried out at the CCR genomics core at NCI, NIH.

### Jurkat-memCherry-*orf-I* cells

The pSDM-N26(p8)-HA or pSDM-G29S(p12)-HA lentiviral GFP expressing plasmid were cloned into pSDM101^81^ (herafter pSDM) after PCR amplification (p12-PmeI-F: **5**′**-**ATTAGTTTAAACGCCACCATGCTGTTTCGCCTTC-3′ and p12-BamHI-R **5**′**-** ATTAGGATCCCTAGAAGAGGAAAGCCGCGGC-3′) of N26 and G29S from the pME18S p12deltaSL expression plasmid as previously described^35,36^, digested with PmeI and BamH1 restriction enzymes and re-cloned into the pmeI and BamH1 digested pSDM vector. All constructs were verified by sequencing. A 10 cm dish with 60–80% confluent 293FT cells was transfected using PEI transfection reagent (Polyscience Inc) where 400 µl of DMEM without FBS was added 12 µg pSDM, pSDM-N26(p8)-HA or pSDM-G29S(p12)-HA, 8 µg of psPAX-2 (Packaging plasmid, Addgene) and 6 µg pMD2.G (Env, Addgene). Further, 26 µl PEI (2 mg/ml) was diluted in 400 µl of DMEM without FBS before combined with the DNA solution, vortexed and incubated at room temperature for 5 min before added to the 10 cm dish with the 293FT cells in 10 ml complete medium. The cells were then incubated for 8 h before the medium was replaced with 8 ml fresh complete medium and cultured for another 48 h.

The cell culture supernatant from the 293FT cells was centrifuged for 5 min at 1500 RPM and then filtered through a 0.22 µm filter. This was followed by centrifugation at 8000 RCF (Eppendorf centrifuge 5418R) for 3 h at 4°C and removal of all supernatant, except 20 µl. The concentrated virus was resuspended in 100 µl of RPMI-1640 without FBS. The Jurkat-memCherry cells were centrifuged at 1200 RPM for 5 min, resuspended in the virus solution, incubated for 5 min at 37°C before centrifuged for 10 min at 800 RCF. Finally, the cells were resuspended in 1 ml of complete medium in a 12-well plate for 72 h. Transduction efficiency was measured by flow cytometry (FACSCalibur, BD).

### Generation and characterization of HTLV-1 primary human cells: CD4^+^-pAB-D26

Stable HTLV-1 producing 729.6 human lymphoblastoid B-cells were generated as previously described^37^. The 729–6 B-cell line infected with the pAB wild-type virus were maintained in RPMI 1640 with 10% FBS. Using negative selection beads (StemCell), CD4^+^ T-cells were isolated from healthy donors peripheral blood mononuclear cells. Stable HTLV-1 producing CD4^+^ T-cell lines were established by co-cultivation of donor uninfected primary HLA.A2+/CD4^+^ T-cells with lethally γ-irradiated 729.6-HTLV-1 infected cells. T-cells were cultured in RPMI supplemented with 20% FBS and 100 U of interleukin-2 for several months. Viral genomic sequences verified by sequencing of the ClaI-SalI fragment as described previously^36^.

### Phenotyping of CD4^+^ HTLV-1 cells

CD4^+^-pAB-D26 HTLV-1 cells, were stained with the antibodies anti-CD3 (Alexa Fluor 700), anti-CD4 (peri- dinin chlorophyll protein [PerCP]-Cy5.5), anti-CD8 (Qdot 655), anti-CD20 (Qdot 605), all obtained from BD Biosciences and additionally stained with LIVE/DEAD fixable aqua dead cell stain (Molecular Probes).

### DNA extraction

CD4^+^ HTLV-1 cells were submitted to genomic DNA extraction. Genomic DNA was isolated using a DNeasy blood and tissue kit (Qiagen). PCR amplification was performed with 100 ng of DNA with the following primers gag-F1 (5-GGCCAAATCCTTTCC CGTAG-3) and gag-R1 (5′-GTTGTGGATTGTTGGCTTGG-3′), β-actin-F1 (5′-CGGTTGGCCTTGGGGTTCAGGGGG-3′) and β-actin-R1 (5′-ATCGTGGGGCGCCCCAGGCACCA-3′). Correctly sized amplicons were identified by 1% agarose gel electrophoresis.

### Quantitation of HTLV-1 proviral DNA

HTLV-1 proviral load was measured in CD4^+^ HTLV-1 T cells by quantitative realtime PCR using TaqMan probe, as described previously^82^ with slight modifications. Genomic DNA was extracted with QIAGEN blood mini kit (QIAGEN) and subjected to PCR. Copy number of HTLV-1 was measured with primers for pX region of HTLV-1. RNase P gene, detected with TaqMan RNase P Control Reagents Kit (Applied Biosystems), was used as the endogenous reference in multiplex reactions. The proviral load (%) was shown as the copy number of HTLV-1 per 100 of copy number of RNase P gene.

### Microscopy

The fluorescent microscope AxioObserver Z1, Delta Vision Elite wide-field deconvolution microscope and the LSM780 confocal microscope (Carl Zeiss, Inc, Thornwood, NY) were used. Alpha Plan Apochromat 63X/1.4 NA Oil DICIII and appropriate filter sets for AxioObserver Z1 and Alpha Plan Apochromat 63X/1.46 NA Oil. Lasers: 25 mW Argon 488, 20 mW Diode Laser 561 nm for LSM780. Images were analyzed by the ZEN 2012 software. For scanning electron microscopy, images were captured by Jeol JSM-7400F LEI 4.0 kV, x3000 (x3700 for D2) and WD 8.0 mm. Figures were generated using Photoshop CS6 and Zen software (Carl Zeiss). For 3D construction ImageJ 1.51k (National Institutes of Health (NIH)) was used on Z-stacks (1 µm slices) using default settings for the 3D project plugin, including brightest point as projection method with Y-axis rotation. Images were prepared using Adobe Photoshop CS6 and Adobe Illustrator CS6.

### Statistical analysis

Unpaired t-test was performed to evaluate statistic significant changes. F-test was performed to verify that the individual variations were not significant. GraphPad Prism (Version 6.03) was used (*p ≤ 0.05, **p ≤ 0.01, ***p ≤ 0.001).

## Electronic supplementary material

Supplementary Information

Supplementary Video 1

Supplementary Video 2
