# Supplementary material for: Inhibition of Tunneling Nanotube (TNT) Formation and Human T-cell Leukemia Virus Type 1 (HTLV-1) Transmission by Cytarabine
Source: Sci Rep. 2018 Jul 24;8:11118. doi: 10.1038/s41598-018-29391-w (PMC6057998; doi:10.1038/s41598-018-29391-w)
Supplement: Supplementary file 1 — Supplementary Information [file 41598_2018_29391_MOESM1_ESM.pdf]

Supplementary Information for

Inhibition of Tunneling Nanotube (TNT) Formation and Human T-cell Leukemia Virus Type 1 (HTLV-1) Transmission by Cytarabine

Maria Omsland, Cynthia Pise-Masison, Dai Fujikawa, Veronica Galli, Claudio Fenizia, Robyn Washington Parks, Bjørn Tore Gjertsen, Genoveffa Franchini and Vibeke Andresen*

*Corresponding author: Vibeke.andresen@uib.no

Supplementary Figure 1

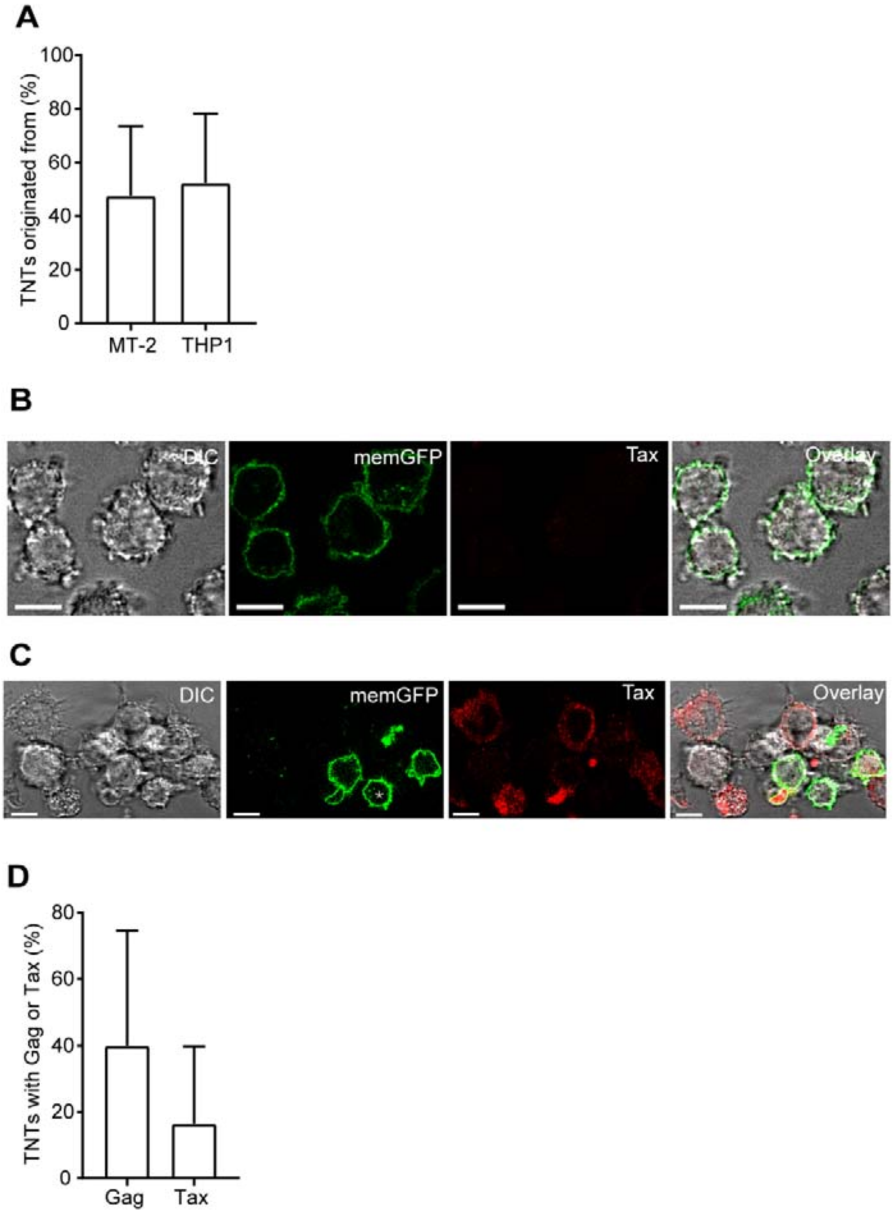

Supplementary Figure 1. Quantification of origin and presence of viral proteins in the TNTs and specificity of the anti-Tax antibody. (**A**) Quantification of the cells generating TNTs between THP-1 (mem-GFP) and MT-2. Images of a total of 33 TNTs from five independent experiments were used for the quantification. (**B**) Immunofluorescence of THP-1 (mem-GFP) cells stained with the Tax-tab172 antibody and Alexa-fluor-568 as secondary

antibody. Image is representative of two independent experiments. (**C**) Co-culture of 14 000 THP-1 (mem-GFP) cells and 56 000 MT-2 cells stained with Tax-tab172 with Alexa fluor 568 as secondary antibody 24h after co-culture. Star indicates a Tax-negative THP-1 (mem-GFP) cell. Image is representative from two independent experiments. (**D**) Quantification of the presence of Tax or Gag in TNTs from (A). A total of 14 TNTs from two independent experiments was counted for Tax presence and 20 TNTs from three independent experiments was used for the quantification of Gag presence.

Supplementary Figure 2

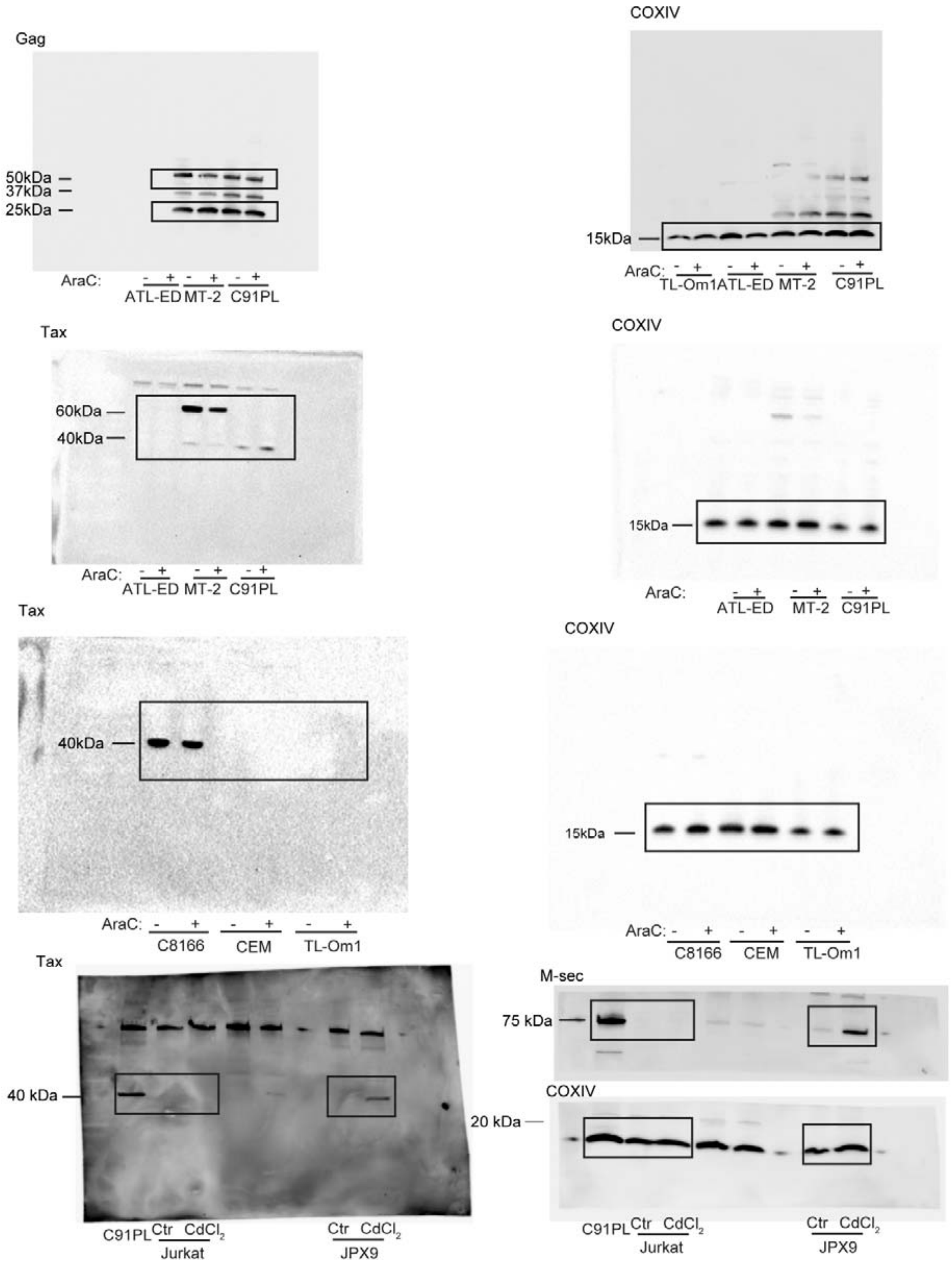

Supplementary figure 2. Uncropped immunoblots from figure 5D, E and F.

Supplementary Figure 3

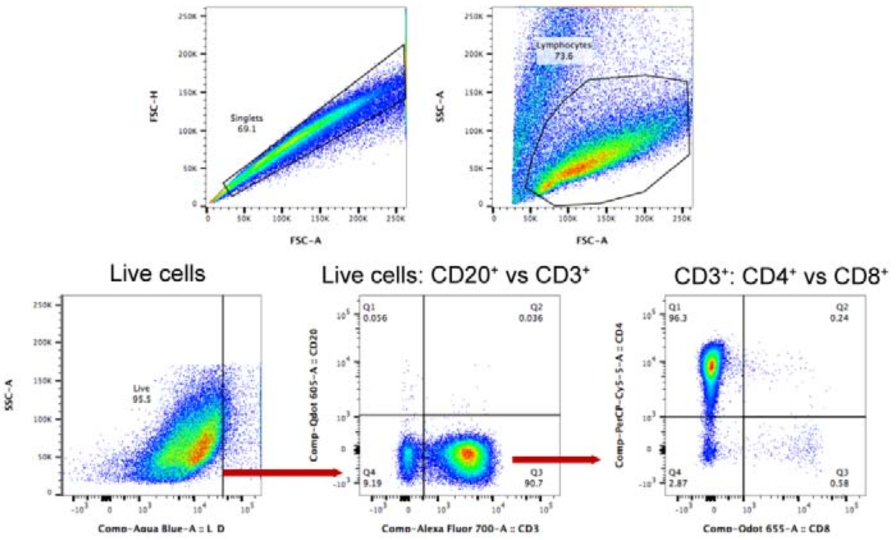

Supplementary Figure 3. Verification of T-cell markers in primary CD4$^+$ cells after co-culture with irradiated 729.6 cells pAB-D26. The cells were fixed and stained with antibodies against CD3, CD4, CD8 and CD20 and analyzed by flow cytometry. Scatterplot of the gating strategy is shown. Majority of the cell population after 7 days in culture express CD3$^+$ and CD4$^+$, but is negative for CD8 and the B-cell marker CD20.

Supplementary Figure 4

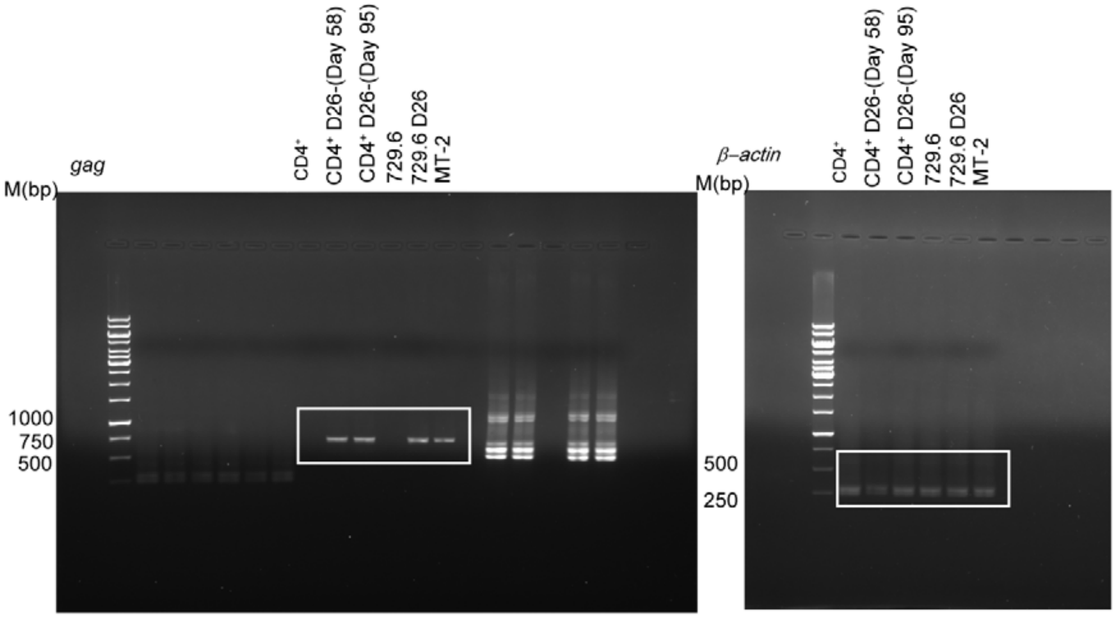

Supplementary figure 4. Uncropped gel from figure 6D.

Supplementary Figure 5

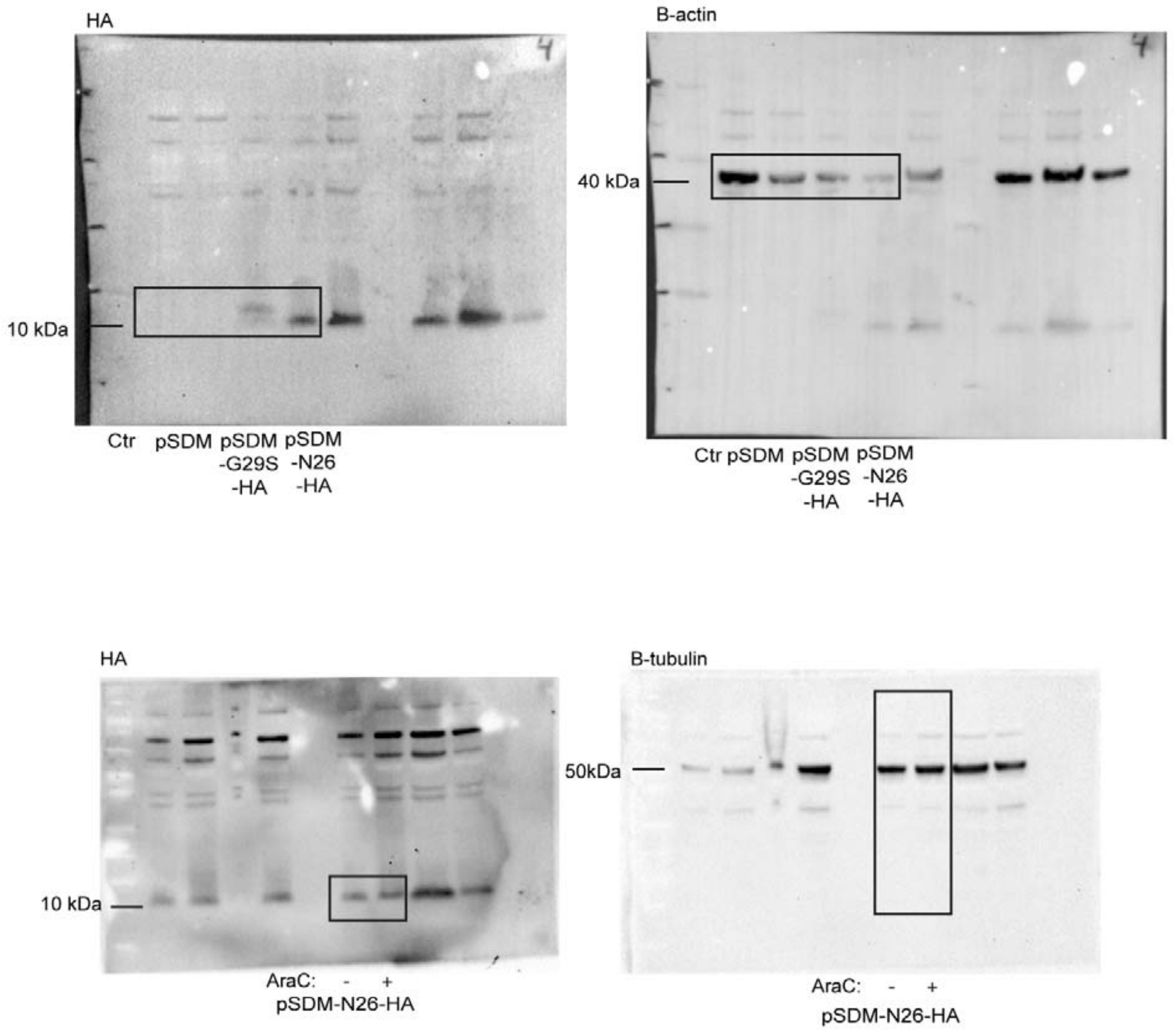

Supplementary figure 5. Uncropped immunoblots from figure 7C and G.

Supplementary Video Legends

Supplementary video 1. A 3D reconstruction of Z-stack images from Figure 3C. Co-culture of THP-1 (mem-GFP) and MT-2 cells (24h) stained with Tax-tab172 and Alexa fluor 568 as secondary antibody. 3D construction was performed using 3D plug-in in ImageJ. Image is representative from two independent experiments. Scale bar 10 µm.

Supplementary video 2. A 3D reconstruction of Z-stack images from Figure 4A. Co-culture of THP-1 (mem-GFP) and MT-2 cells (24h) stained with anti-Gag and Alexa fluor 568 as secondary antibody. 3D construction was performed using 3D plug-in in ImageJ. Image is representative from three independent experiments. Scale bar 10 µm.
